# Intrinsic resistance to terbinafine among human and animal isolates of *Trichophyton mentagrophytes* related to amino acid substitution in the squalene epoxidase

**DOI:** 10.1007/s15010-020-01498-1

**Published:** 2020-08-08

**Authors:** Dominik Łagowski, Sebastian Gnat, Aneta Nowakiewicz, Marcelina Osińska, Mariusz Dyląg

**Affiliations:** 1 Department of Veterinary Microbiology, Faculty of Veterinary Medicine, Institute of Preclinical Veterinary Sciences, University of Life Sciences, Akademicka 12, 20-033 Lublin, Poland; 2 Department of Mycology and Genetics, Faculty of Biological Sciences, Institute of Genetics and Microbiology, University of Wroclaw, Wroclaw, Poland

**Keywords:** *Trichophyton mentagrophytes*, Squalene epoxidase, Terbinafine, Antifungal resistance

## Abstract

**Background:**

Dermatomycoses are the most common fungal infections in the world affecting a significant part of the human and animal population. The majority of zoophilic infections in humans are caused by* Trichophyton mentagrophytes*. Currently, the first-line drug for both oral and topical therapy is terbinafine. However, an increasing number of cases that are difficult to be cured with this drug have been noted in Europe and Asia. Resistance to terbinafine and other allylamines is very rare and usually correlated with point mutations in the squalene epoxidase gene resulting in single amino acid substitutions in the enzyme, which is crucial in the ergosterol synthesis pathway.

**Purpose:**

Here, we report terbinafine-resistant* T. mentagrophytes* isolates among which one was an etiological factor of tinea capitis in a man and three were obtained from asymptomatic foxes in Poland.

**Methods:**

We used the CLSI protocol to determine antifungal susceptibility profiles of naftifine, amphotericin B, griseofulvin, ketoconazole, miconazole, itraconazole, voriconazole, and ciclopirox. Moreover, the squalene epoxidase gene of the terbinafine-resistant strains was sequenced and analysed.

**Results:**

In the genomes of all four resistant strains exhibiting elevated MICs to terbinafine (16 to 32 µg/ml), single-point mutations leading to Leu393Phe substitution in the squalene epoxidase enzyme were revealed. Among the other tested substances, a MIC50 value of 1 µg/ml was shown only for griseofulvin.

**Conclusion:**

Finally, our study revealed that the terbinafine resistance phenomenon might not be acquired by exposure to the drug but can be intrinsic. This is evidenced by the description of the terbinafine-resistant strains isolated from the asymptomatic animals.

## Introduction

Fungal infections of skin, hairs, and nails are the most prevalent mycoses worldwide with a high economic burden, as approximately $1.67 billion is spent on treatment each year [1–3]. The main etiological factors of superficial mycoses are dermatophytes, which are a cosmopolitan group encompassing more than 50 species classified in the genera *Trichophyton, Microsporum, Epidermophyton, Arthroderma, Nannizzia, Lophophyton*, and *Paraphyton* [4, 5]. The sources of dermatophytes include the natural environment, i.e., soil (geophilic species), and transmission via direct or indirect contact with infected humans (antropophilic species) or animals (zoophilic species) as well as asymptomatic carriers [6, 7]. Improper hygiene, occlusive footwear, socioeconomic conditions, profession, animal breeding, diabetes mellitus, age, genetics, and immunocompromised status can increase the likelihood of infection [3, 8–11].

Currently, there are numerous options for the treatment of dermatophyte infections. Similar antifungal treatments are used worldwide for the most of them; however, there are some variations and country-specific guidelines should be consulted [1, 9]. Most superficial infections caused by dermatophytes are successfully treated with terbinafine [1, 12]. This antimycotic belongs to the allylamine group and is recommended as the first-line oral medication for the treatment of such infections [13–15]. The drug disturbs the formation of ergosterol, i.e., the major sterol of the fungal membrane, by blocking the squalene epoxidase enzyme and subsequently inhibiting the fungal growth [16, 17].

In the first decade of the twenty-first century, resistance to terbinafine among dermatophytes was found to be rare and primarily limited to *Trichophyton rubrum* clinical isolates [18, 19]. Recently, more and more cases can be found in the literature, which are difficult to treat using this substance [20, 21]. Moreover, reports from Asian and European countries indicate that microbial resistance to terbinafine is revealed in other dermatophyte species, i.e., *Trichophyton interdigitale* and *Trichophyton mentagrophytes* [21–28]. Interestingly, the molecular mechanism underlying the reduced susceptibility to terbinafine is usually correlated with nonsynonymous point mutations in the squalene epoxidase (*SQLE*) gene [27–29].

In view of the incidence of patients with dermatomycoses that are insensitive to terbinafine treatment, the aim of this study was to perform antifungal susceptibility testing of allylamine drugs, compared to other groups of antifungals, in *Trichophyton mentagrophytes* clinical isolates obtained from humans and animals, and dermatophytes from asymptomatic carriers. Moreover, the squalene epoxidase (*SQLE*) gene amplified based on genomic DNA isolated from the terbinafine-resistant strains was sequenced and analysed for comparison with reference sequences available in the GenBank database.

## Materials and methods

### Dermatophyte strains

**Fig. 1:**
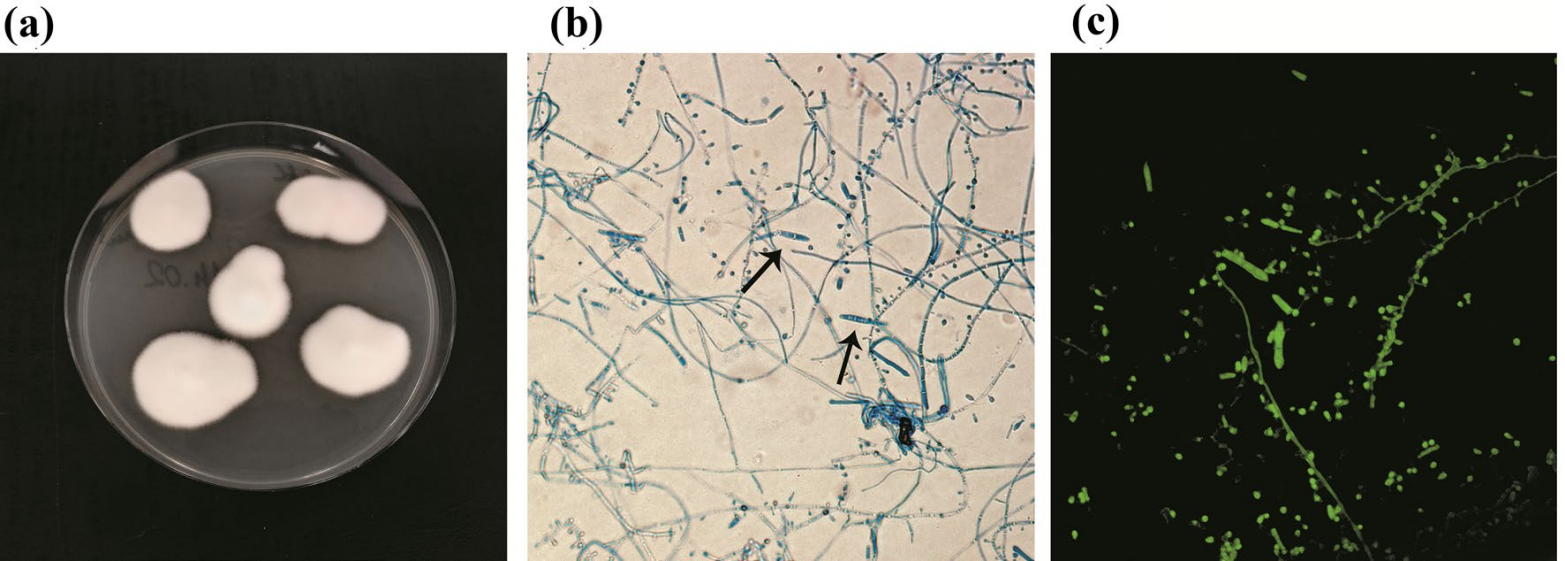
Micro- and macroscopic morphology of *Trichophyton mentagrophytes* isolated after 20 days of incubation (Olympus BX51, Tokyo, Japan). Flat, white colonies with a powdery surface; numerous single-celled, clavate microconidia located laterally to the hyphae; multicelled macroconidia in the shape of a cigar; **a** obverse on Sabouraud medium; **b** micromorphology in light microscopy at 400 × , stained with lactophenol blue; **c** micromorphology in fluorescence microscopy at 400 × , stained with calcofluor white

In total, 29 clinical isolates of *Trichophyton mentagrophytes* obtained from patients with dermatophytosis (*n* = 7), symptomatic animals (*n* = 15), and asymptomatic animal carriers (*n* = 7) were obtained from different regions of Poland (Fig. 1). The dermatophyte strains were obtained from clinical cases of zoophilic origin infections in humans, outbreaks of symptomatic mycoses in animals, and asymptomatic animals that have never been diagnosed with dermatophytosis. Isolates were collected between 2016 and 2019. All isolates were identified to the species level by a combination of macro- and micro-morphology examination and the internal-transcribed spacer (ITS) rDNA region sequencing technique [30]. DNA was isolated from the dermatophytes with the phenol–chloroform method [31]. All clinical isolates were deposited in the culture collections of the Department of Veterinary Microbiology, University of Life Sciences in Lublin, Poland, and the nucleotide sequences—in the GenBank database (Table 1).

**Table 1:** Isolates of dermatophytes obtained from symptomatic and asymptomatic animals, and humans with description

Isolates	Host	Isolation source	Accession numbers of ITS sequences	Accession numbers of SQLE sequences	Amino acid substitution in SQLE gene
TMA10	Fox	Asymptomatic	MT106082	MT159953	Leu393Phe
TMA11	Fox	Asymptomatic	MT106083	MT130520	None
TMA12	Guinea pig	Asymptomatic	MT106084	–	–
TMA13	Chinchilla	Asymptomatic	MT106085	–	–
TMA14	Fox	Asymptomatic	MT106086	MT159954	Leu393Phe
TMA15	Fox	Asymptomatic	MT106087	MT159955	Leu393Phe
TMA16	Guinea pig	Asymptomatic	MT106088	MT130524	None
TMS20	Fox	Clinical lesions	MT106062	MT130521	None
TMS21	Fox	Clinical lesions	MT106063	MT130522	None
TMS22	Fox	Clinical lesions	MT106064	MT130523	None
TMS23	Cat	Clinical lesions	MT106065	MT130527	None
TMS24	Guinea pig	Clinical lesions	MT106066	MT130525	None
TMS25	Guinea pig	Clinical lesions	MT106067	–	–
TMS26	Dog	Clinical lesions	MT106068	–	–
TMS27	Dog	Clinical lesions	MT106069	MT130526	none
TMS28	Cat	Clinical lesions	MT106070	–	–
TMS29	Fox	Clinical lesions	MT106071	–	–
TMS30	Fox	Clinical lesions	MT106072	–	–
TMS31	Fox	Clinical lesions	MT106073	–	–
TMS32	Cat	Clinical lesions	MT106074	–	–
TMS33	Guinea pig	Clinical lesions	MT106075	–	–
TMS34	Guinea pig	Clinical lesions	MT106076	–	–
TMH1	Human	Tinea capitis	MT106055	MT130516	None
TMH2	Human	Tinea capitis	MT106056	MT130517	None
TMH3	Human	Tinea capitis	MT106057	MT130518	None
TMH4	Human	Tinea unguium	MT106058	MT130519	None
TMH5	Human	Tinea capitis	MT106059	–	–
TMH6	Human	Tinea capitis	MT106060	–	–
TMH7	Human	Tinea capitis	MT106061	MT156570	Leu393Phe

*NCBI* National Center for Biotechnology Information

### Antifungal drug susceptibility tests

In vitro susceptibility testing of allylamine, polyene, imidazole, triazole, and pyridinone derivatives drugs was performed according to the Clinical and Laboratory Standards Institute (CLSI) document M38-A2 [32]. Reagent-grade amphotericin B (AMB), ciclopirox (CPO), griseofulvin (GRE), itraconazole (ITC), ketoconazole (KTC), miconazole (MCZ), naftifine (NFT), terbinafine (TRB), and voriconazole (VRC) were obtained in the powder form. Drug stock solutions were prepared in dimethyl sulfoxide (DMSO) to reach the final DMSO concentration in the wells below 1%. The drugs were analysed at the final concentration comprised in the range of 0.001–32 μg/ml. The dermatophyte isolates were cultured on potato dextrose agar (PDA; Difco) for 21 days, and conidial suspensions were prepared by gentle scraping mature colonies into sterile physiological saline containing 0.002% Tween 80. Homogeneous inoculum supernatants were collected, and their optical density (OD) at 530 nm was adjusted spectrophotometrically to transmission ranged from 65 to 70%, and the final density of inoculum was 1 × 10^3^ to 3 × 10^3^ CFU/ml. The inocula were diluted 1:50 in RPMI 1640 medium and incubated with the indicated concentrations of the antifungals in 96-well plates at 30 °C for 72 h. Minimum inhibitory concentrations (MICs) were determined visually using a reading mirror. All tests were performed in triplicate, and differences between mean values were assessed by Student’s *t* test using the R program. All the compounds used in the present experiments were purchased from Sigma-Aldrich (Missouri, USA) if not stated otherwise. In addition, verification of the terbinafine-resistant isolates was based on fungal growth on Sabouraud glucose agar (SGA, Biomaxima, Lublin, Poland) containing 0.2 µg/ml of this substance [22]. Examination of *T. mentagrophytes* growth was performed after 7, 10, and 14 days.

### Squalene epoxidase (SQLE) gene sequencing and analysis

Partial squalene epoxidase gene sequences in the case of four resistant and twelve susceptible isolates were analysed with few modifications as previously described by Singh et al. [21]. The *SQLE* gene amplification reaction was carried out in a T Personal thermal cycler (Biometra GmbH, Göttingen, Germany) with 25 µl of the reaction mixture composed of 12.5 µl Qiagen Taq PCR Master Mix (Qiagen, Hilden, Germany), 10 pmol of each primer: Tr*SQLE*-F1 (5′-ATGGTTGTAGAGGCTCCTCCC-3′) and Tr*SQLE*-R1 (5′-CTAGCTTTGAAGTTCGGCAAA-3′), and 1 µl of DNA template for 30 cycles consisting of template denaturation (1 min, at 95 °C), primer annealing (30 s, at 55 °C), and elongation (3 min, at 72 °C). The PCR products were separated on 2% agarose gel stained with ethidium bromide and visualised. The *SQLE* gene sequencing reaction was carried out using a BigDye Terminator Cycle Sequencing Kit (Life Technologies, Carlsbad, California, USA) and primers Tr*SQLE*-F1 and Tr*SQLE*-R1. The PCR mixture (10 µl) contained 2 µl of 2.5 × concentrated Ready Reaction Premix, 1 µl of 5 × concentrated BigDye Sequencing Buffer, 0.25 µl of the primer at a concentration of 5 pmol (initially 100 pmol), a DNA amplicon at a concentration of 50 ng, and sterile distilled water at a final volume of 10 µl. Two separate reactions were carried out for primers Tr*SQLE*-F1 and Tr*SQLE*-R1. PCR was performed in a T Personal cycler (Biometra GmbH) with the following conditions: initial denaturation for 1 min at 96 °C, denaturation for 10 s at 96 °C, annealing of primers for 5 s at 50 °C, and elongation for 4 min 60 °C. The final three stages, i.e., denaturation, annealing of primers, and elongation, were repeated 25 times. The PCR product was purified using an ExTerminator kit (A&A Biotechnology, Gdynia, Poland) and then the DNA sequence was read in a 3500 Genetic Analyser from Life Technologies (Carlsbad, California, USA). The nucleotide and predicted amino acid sequences of the *SQLE* gene in all the *T. mentagrophytes* isolates tested were compared with the reference sequences available in the GenBank database.

## Results

The MIC ranges, MIC_GM_, MIC_50_, and MIC_90_ ratios of the nine antifungal drugs tested on the pool of 29 *T. mentagrophytes* isolates obtained from humans, symptomatic, and asymptomatic animals are summarized in Table 2. Terbinafine exhibited the lowest MIC_50_ and MIC_90_ values in comparison with the other drugs, whereas four highly resistant isolates that were found resulted in the highest MIC_GM_ value of this substance for all isolates. Griseofulvin was found to exert the weakest in vitro effect and had the highest MIC_50_, MIC_90_, and Mode values. Additionally, naftifine, griseofulvin, and miconazole had the widest MIC range, i.e., 0.125–4 μg/ml, 0.125–4 μg/ml, and 0.03–12 μg/ml, respectively. Remarkably, the MIC_50_, MIC_90_, and Mode values of terbinafine, amphotericin, ketoconazole, miconazole, itraconazole, voriconazole, and ciclopirox against all *T. mentagrophytes* isolates were below 1 μg/ml, whereas those of griseofulvin and MIC_90_ of naftifine were above 1 μg/ml. The verification test of resistance of the isolates to terbinafine on Sabouraud's medium supplemented with 0.2 μg/ml of this substance confirmed the presence of resistant strains in four cases.

**Table 2:** In vitro antifungal susceptibilities of 29 clinical isolates of *Trichophyton mentagrophytes* obtained from symptomatic and asymptomatic dermatophytosis

Antifungal agents		Host	MIC (µg/ml)														MIC range	MIC_50_	MIC_90_	MIC_GM_	Mode
			0.004	0.008	0.016	0.03	0.06	0.125	0.25	0.5	1	2	4	8	16	32					
Allylamine	NFT	humans								1	2	3	1**				0.125–4	0.5	2	1.03	0.5
		asymptomatic animals							2	2	3										
		symptomatic animals							6	5	1	2**	1**								
	TRB	humans	4	2												1*	0.004–32	0.004	0.016	2.21^ST^	0.004
		asymptomatic animals	3	1											3*						
		symptomatic animals	11	3	1																
Polyenes	AMB	humans							4	3							0.125–1	0.25	0.5	0.44	0.25
		asymptomatic animals						2	5												
		symptomatic animals						2	8	4	1										
	GRE	humans								1	3	3					0.125–4	1	1	1.05	1
		asymptomatic animals							1	2	2	1	1								
		symptomatic animals						1	2	4	7	1									
Imidazoles	KTC	humans					1	2	2	1	1						0.06–1	0.25	0.5	0.39	0.25
		asymptomatic animals					3	2	1	1											
		symptomatic animals						5	8	1	1										
	MCZ	humans				1		3	2	1							0.03–1	0.125	0.5	0.2	0.125
		asymptomatic animals					5	2													
		symptomatic animals						9	3	2	1										
Triazoles	ITC	humans			2	2	3										0.008–0.06	0.03	0.06	0.03	0.03
		asymptomatic animals				6	1														
		symptomatic animals		3	5	7															
	VRC	humans				7											0.016–0.25	0.03	0.125	0.05	0.03
		asymptomatic animals				4	2	1													
		symptomatic animals			6	5	2	1	1												
Pyridinone derivatives	CPO	humans				2	2	3									0.008–0.125	0.03	0.125	0.04	0.03
		asymptomatic animals			2	5															
		symptomatic animals		2	4	7	1	1													

*AMB* amphotericin B, *CPO* ciclopirox, *GRE* griseofulvin, *ITC* itraconazole, *KTC* ketoconazole, *MCZ* miconazole, *NFT* naftifine, *TRB* terbinafine, *VRC* voriconazole

^*^strains with mutations in squalene epoxidase gene (Leu393Phe)

^**^strains with proven terbinafine resistance

^ST^statistically significantly higher result

**Fig. 2:**
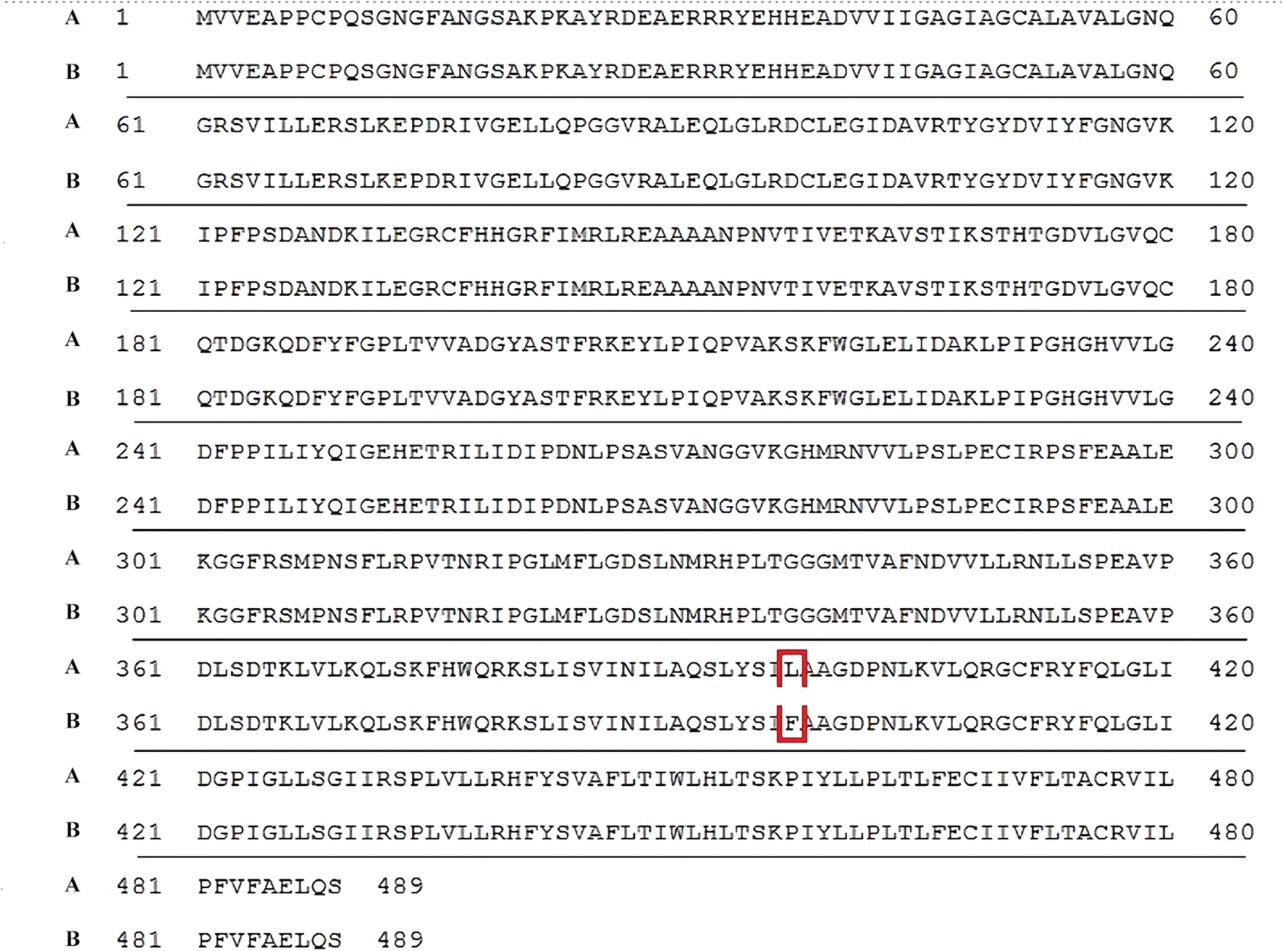
Alignment of squalene epoxidase amino acid sequences. The Leu393Phe substitution is marked in the frame, **a** The amino acid sequence of a terbinafine-resistant strain TMH7 derived from the case of human *tinea capitis* (accession number of nucleotide sequence: MT156570); **b**. amino acid sequence of the reference strain with wild phenotype (*Trichophyton mentagrophytes* NCCPF: 800025; accession number of protein sequence: ATA67033, and nucleotide sequence: KX906454)

The partial sequences of the *SQLE* gene obtained for the terbinafine-resistant strains and selected representatives of other *T. mentagrophytes* strains associated with different hosts were deposited in GenBank (Table 1). Terbinafine-resistant strains were obtained from one case of human tinea capitis (MIC = 32 μg/ml) and three asymptomatic silver foxes (*Vulpes vulpes* L., MIC = 16 μg/ml). All four resistant *T. mentagrophytes* isolates harboured missense mutations in the squalene epoxidase (*SQLE*) gene, corresponding to the same amino acid substitution Leu393Phe (Fig. 2). On the other hand, the susceptible isolates exhibited wild-type *SQLE* sequences, without mutations manifested in changes in the amino acid sequence of squalene epoxidase.

## Discussion

In the past few years, superficial infections caused by filamentous fungi, especially dermatophytes, along with a concomitant increase in the number of difficult-to-treat cases have increasingly been recognized worldwide, becoming a serious public health problem [3, 21, 24, 25]. Moreover, the in vitro drug resistance of fungi observed over the past decade has been alarming, and it seems that elucidation of the underlying molecular mechanisms of this phenomenon is indispensable for successful therapies [3, 22, 27, 33].

Remarkably, in the present study, a considerably high terbinafine resistant rate with approximately 14% was observed among 29* T. mentagrophytes* isolates obtained from infected humans and animals and asymptomatic carriers. In scientific literature, terbinafine has been reported to be the most effective antifungal agent against *Trichophyton* spp. isolates worldwide [19, 34, 35]. Although there are no well-established guidelines for the dosage and duration of systemic therapy in patients with fungal infections [36], terbinafine should be the first-line treatment, as itraconazole is more prone to adverse effects [37]. However, to the best of our knowledge, terbinafine resistance in *T. mentagrophytes* isolates has already been reported in Asian and European countries, including India [21, 23, 25, 26], Switzerland [22, 27], Japan [24], Finland [38], Denmark [28], Bahrain [29], Iran [39], and Russia [40] (Fig. 2). Moreover, the prevalence of terbinafine-resistant clinical isolates of *T. mentagrophytes* ranged from less than 1% in Switzerland [22] to more than 70% in India [26]. In the latter case, the MIC values of terbinafine in resistant isolates varied in the range ≥ 1 – ≥ 32 μg/ml [21, 23, 25, 26]. In our study, the MIC values for the terbinafine-resistant strains were in the range of 16–32 μg/ml. Interestingly, in three cases noted by us, in vitro resistance to terbinafine has been demonstrated for isolates obtained from the asymptomatic foxes. This may be indicative of persistence of terbinafine-resistant strains in hairs of natural animal hosts, which serve as their carriers. Yamada et al. [22] revealed that the high frequency of terbinafine-resistant strains described in the recent years can be explained by the fact that treatment with such a popular drug as terbinafine involves prolonged exposure to the antifungal drug, which could favour the selection of resistant strains. In contrast, Mukharjee et al. [19] concluded that the terbinafine resistance phenomenon might not be acquired via exposure to the drug but can be innate. The detection of terbinafine-resistant strains isolated from the asymptomatic carriers in our study can lead to the same conclusion. Further molecular research of dermatophyte isolates from human and animal infections may provide more insight in delineating the issue of the primary or acquired mechanism of terbinafine resistance.

**Fig. 3:**
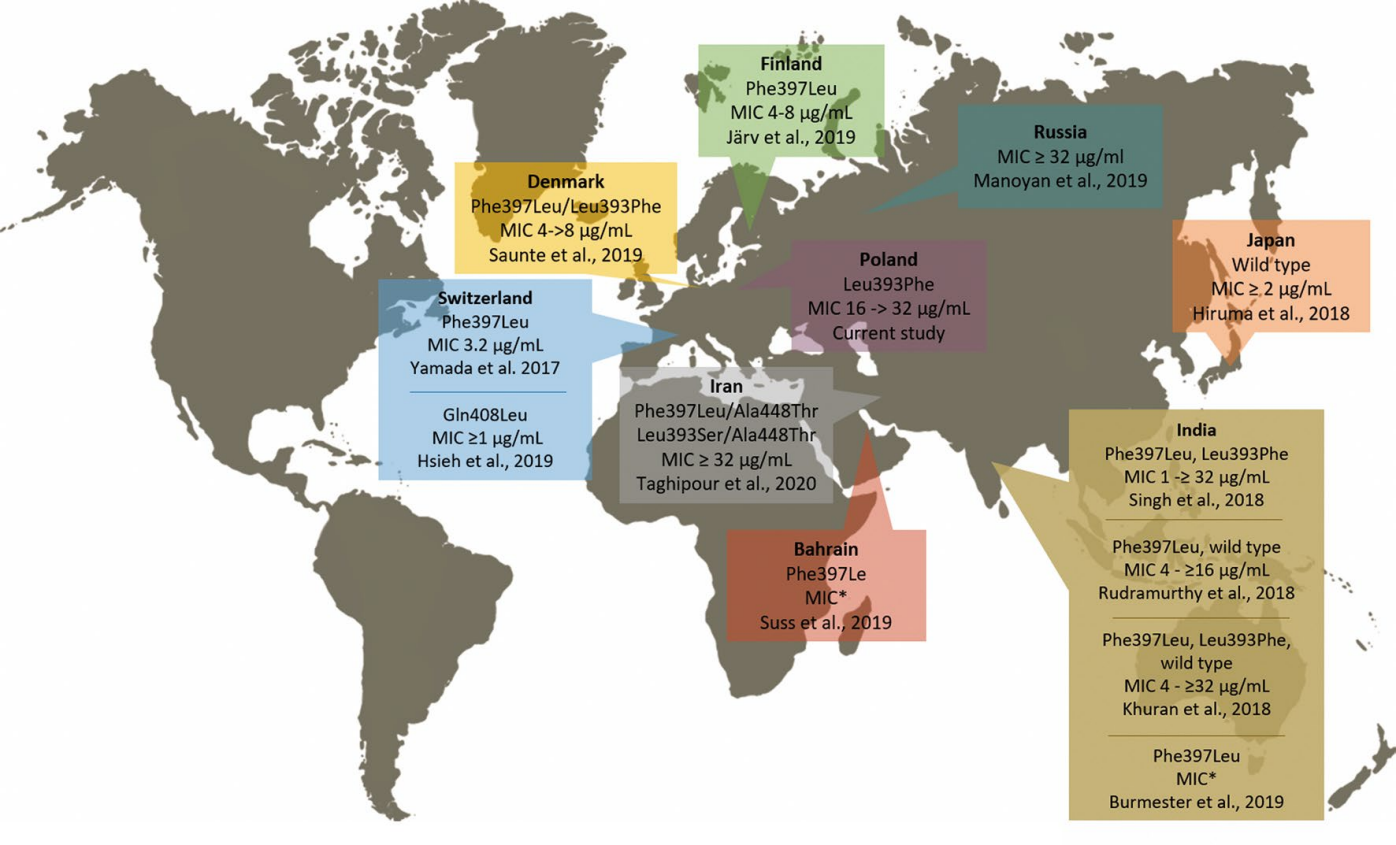
Diagram of geographical locations in which terbinafine resistance of isolates has been described with MICs and substitutions in the squalene epoxidase enzyme

Terbinafine inhibits squalene epoxidase in a non-competitive manner by blocking the synthesis of 2,3-oxidosqualene, leading to accumulation of squalene and depletion of ergosterol, thereby causing growth inhibition [15, 41]. Precise identification of substitutions in the amino acid chain that are responsible for the resistance to this drug is a helpful tool in the determination of the interaction between the antifungal and its target [3, 22, 33]. In this aspect, many recent scientific reports have shown that Phe397Leu [21, 22, 26, 28, 29, 38] and Leu393Phe [21, 25, 28] are the most frequent amino acid substitutions in the squalene epoxidase protein leading to terbinafine resistance (Fig. 3). Furthermore, the Leu393Phe and Phe397Leu substitutions were also reported in the amino acid sequences of *Trichophyton interdigitale* and *Trichophyton rubrum* isolates [18, 19, 21, 23]. Additionally, the Gln408Leu, Leu393Ser, or other less common substitutions were also correlated with high MIC values (≥ 32 μg/ml) of terbinafine [27]. Previously, Leu398Phe and Phe402Leu substitutions were observed in the case of terbinafine resistance in *Candida albicans* and *Saccharomyces cerevisiae* [18, 42]. Interestingly, Yamada et al. [22] revealed that introduction of Leu393Phe and Phe397Leu amino acid substitutions into a terbinafine-sensitive *Arthroderma vanbreuseghemii* strain resulted in 8–512-fold reduction of susceptibility to this substance. In other cases, in vitro terbinafine resistance could not be explained by the presence of any mutation in squalene epoxidase gene sequences [23, 24]. Therefore, other mechanisms than that described herein should still be considered as alternatives for terbinafine treatment failure, i.e., multiplication of the salicylate 1-monooxygenase (*salA*) gene [43].

As demonstrated in the literature, the sensitivity of *T. mentagrophytes* to antifungal substances appears to be dependent on the geographical region from which strains are isolated. Bhatia et al. [44] revealed that *T. mentagrophytes* strains identified in northern India showed low MICs to itraconazole and ketoconazole in comparison to terbinafine (MIC_50_: 0.125 µg/ml for itraconazole, 0.0625 µg/ml for ketoconazole, and 0.5 µg/ml for terbinafine). In turn, clinical isolates of this species obtained in Brazil demonstrated low MICs to terbinafine (MIC_50_ = 0.06 µg/ml) in comparison with griseofulvin (MIC_50_ = 0.5 µg/ml) and itraconazole (MIC_50_ = 0.125 µg/ml) [45]. In this study, the MIC_50_ and MIC_90_ reported for itraconazole, voriconazole, and ciclopirox were found to be relatively low (< 0.125 µg/ml). Our results also indicated that the in vitro antifungal activity of naftifine, i.e., another representative of allyloamine drugs next to terbinafine, against the isolates tested was lower than the activity of amphotericin B and other imidazoles, triazoles, and pyridinone derivatives (MIC_GM_ = 1.03 μg/ml and MIC_50_ = 0.5 μg/ml), and only slightly higher than for griseofulvin (MIC_GM_ = 1.05 μg/ml and MIC_50_ = 1 μg/ml). Moreover, the MIC ranges for the examined antifungal agents were similar for the human and animal *T. mentagrophytes* isolates, although the human strains showed slightly higher resistance to naftifine. However, the geographical predisposition to differential sensitivity to antifungal drugs should be further analysed.

In conclusion, it seems that the absence of threshold and cut off values of antifungal substances for practical clinical application for dermatophytes results in an increase in the MICs. However, the increase may not always be associated with the mechanism of drug resistance, but rather with the higher dosage of the antifungal or the longer duration of treatment required for an optimal clinical response. In this study, we have revealed that high-level in vitro terbinafine resistance may emerge with the analysed mutations in the squalene epoxidase gene in clinical isolates of *T. mentagrophytes* in both animals and humans. This fact is worrying and necessitates more frequent genotyping of isolates that are primarily resistant to terbinafine.
